# *De Novo* Transcriptome Analysis for Kentucky Bluegrass Dwarf Mutants Induced by Space Mutation

**DOI:** 10.1371/journal.pone.0151768

**Published:** 2016-03-24

**Authors:** Lu Gan, Rong Di, Yuehui Chao, Liebao Han, Xingwu Chen, Chao Wu, Shuxia Yin

**Affiliations:** 1 Institute of Turfgrass Science, Beijing Forestry University, Beijing, 100083, China; 2 Department of Plant Biology and Pathology, Rutgers University, New Brunswick, New Jersey, 08901, United States of America; University of Western Sydney, AUSTRALIA

## Abstract

Kentucky bluegrass (*Poa pratensis* L.) is a major cool-season turfgrass requiring frequent mowing. Utilization of cultivars with slow growth is a promising method to decrease mowing frequency. In this study, two dwarf mutant selections of Kentucky bluegrass (A12 and A16) induced by space mutation were analyzed for the differentially expressed genes compared with the wild type (WT) by the high-throughput RNA-Seq technology. 253,909 unigenes were obtained by *de novo* assembly. 24.20% of the unigenes had a significant level of amino acid sequence identity to *Brachypodium distachyon* proteins, followed by *Hordeum vulgare* with 18.72% among the non-redundant (NR) Blastx top hits. Assembled unigenes were associated with 32 pathways using KEGG orthology terms and their respective KEGG maps. Between WT and A16 libraries, 4,203 differentially expressed genes (DEGs) were identified, whereas there were 883 DEGs between WT and A12 libraries. Further investigation revealed that the DEG pathways were mainly involved in terpenoid biosynthesis and plant hormone metabolism, which might account for the differences of plant height and leaf blade color between dwarf mutant and WT plants. Our study presents the first comprehensive transcriptomic data and gene function analysis of *Poa pratensis* L., providing a valuable resource for future studies in plant dwarfing breeding and comparative genome analysis for Pooideae plants.

## Introduction

Kentucky bluegrass (*Poa pratensis* L.) is a forage crop native to Europe, Asia, North America and northern Africa [1]. It is also one of the most widely used cool-season turfgrasses in temperate and subarctic climates. Compared to other grasses, Kentucky bluegrass is advantageous in excellent tolerance to low temperature, extended drought periods, good spring green-up rate and outstanding recuperative capacity [2]. Frequent mowing (about once a week) is required to keep the preferred mowing height of Kentucky bluegrass to 1.5 to 3 inches for well-maintained turf area [3]. Mowing, as the most basic cultural practice of turf, is labor-intensive and time-consuming. Several plant growth regulators, such as ethephon, trinexapac-ethyl and endothal, have been developed and applied on turf to reduce or slow the growth of grasses, thus decreasing the mowing requirement [3]. An alternative option to decrease mowing frequency is to develop dwarf grass cultivars.

Dwarfism, an important agronomic trait, has been studied extensively on field crops and model plants such as rice (*Oryza sativa* L.), wheat (*Triticum aestivum* L.) and Arabidopsis (*Arabidopsis thaliana* L.) [4–6]. It has been shown that plant dwarf can be resulted from the blocked metabolism and signal transduction pathways of plant hormones, such as gibberellins (GA) or brassinolide (BR) [7], or cell wall and cell elongation [8]. The studies on Arabidopsis, rice and wheat indicate that the genetic variation on synthesis or signal transduction pathways of plant hormones, including GA [9], BR and auxin [10], has a great impact on plant height.

Kentucky bluegrass belongs to the Poaceae family that includes some of the major cereal crops, forage and turf grasses [11]. According to previous comparative genomics analysis, these plants have more homologous genes [12]. However, compared to other plants, the great complexity of Kentucky bluegrass’s genome makes it difficult to fully understand the whole genome [13]. Up to date, only 720 nucleotide sequences of Kentucky bluegrass are available in GenBank, assuming no sequence duplications. The scarcity of genome information is an obstacle to develop new cultivars of Kentucky bluegrass by modern genetic and genomic methods. The recent advances in sequencing technologies have enabled the transcriptomic analysis of many grass species [14]. For non-model species such as Kentucky bluegrass that lacks the sequenced genome, RNA-Seq is a valuable tool for the development of new genetic resources [15]. RNA-Seq technology has been initiated on turfgrass to understand how they respond to biotic and abiotic stresses. For instance, RNA-Seq studies have been done on creeping bentgrass (*Agrostis stolonifera* L.) and *Sclerotinia homoeocarpa* L., the causal agent of dollar spot disease. Several genes that were differentially expressed during the infection were identified from either the host or the pathogen [16]. The transcriptome of two buffalograss (*Buchloe dactyloides* L.) cultivars were sequenced with both the Illumina GA and 454 Titanium FLX sequencing platforms, and a total of 325 differentially expressed genes, which may contribute to the differences between the two cultivars, were identified [17]. Studer *et al*. demonstrated an efficient approach of using next-generation sequencing (NGS) data to construct a high density genetic linkage map based on DNA genomics, and the integration of genetic and physical maps for perennial ryegrass (*Lolium perenne* L.), one of the most important turfgrasses [18]. The high-throughput sequencing was also utilized to characterize the leaf transcriptome of guinea grass (*Panicum maximum* Jacq.), a tropical African grass used for beef cattle feed, leading to the identification of a number of potential molecular markers [19]. To the best of our knowledge, there has been no high-throughput sequencing analysis for Kentucky bluegrass.

Kentucky bluegrass dwarf mutants used in this study were derived from seeds exposed to space environment. Space mutation, one of the physical mutation approaches, has been applied to plant breeding in the past 30 years in China [20]. A number of new cultivars or selections of rice, wheat, maize, green pepper and watermelon were developed by this method [21, 22]. The space mutation approach results in abundant, non-directional mutations [23]. In this study, the two dwarf mutants, A12 and A16, were selected from the offspring of Kentucky bluegrass (cv. Baron) seeds brought to space by the recoverable satellite in 2004. It was noted that the growth rate of dwarf mutants was nearly half of the wild type’s (WT) in the growing season.

To explore the transcriptomic profiles of Kentucky bluegrass dwarf mutants, the high-throughput paired-end Illumina technology was utilized. A total of 45 Gb of read sequences and 253, 909 unigenes were generated from the WT, A12 and A16 in this study. Our objectives were (i) to analyze the differentially expressed genes between Kentucky bluegrass dwarf mutants and WT, (ii) to characterize and annotate expressed unigenes related to physiological characteristics of Kentucky bluegrass, and (iii) to provide a genetic basis for Kentucky bluegrass transcriptomic analysis. As a result, we speculate that the dwarfing of our Kentucky bluegrass may be related to differentially expressed genes in terpenoid metabolic pathway, especially in the GA biosynthetic pathways.

## Materials and Methods

All the study materials were planted in the field of Turfgrass Research Station of Beijing Forestry University, Changping District, Beijing, China. No specific permissions were required for this location/activity because the land is owned by the Institution of Turfgrass Science of Beijing Forestry University. We confirm that the field studies did not involve endangered or protected species.

### Materials and GA application

**Fig 1:**
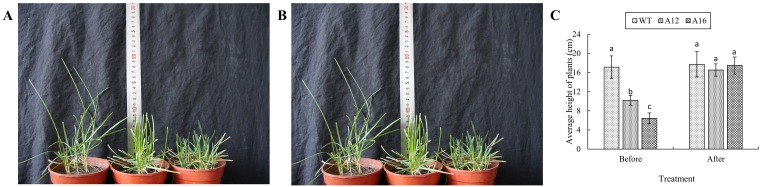
A. WT, A12 and A16 (from left to right) plants without GA_3_ spraying. B. WT, A12 and A16 (from left to right) plants 7 days after foliar-spraying with GA_3._ C plant height comparison between WT, A12 and A16 before and after GA_3_ spraying. Vertical bars on the top indicate standard deviation, and bars with different letters indicate significant difference (*p* < 0.05).

Dry seeds of Kentucky bluegrass (cv. Baron) were brought to the space by No. 20 Recoverable Science &Technology Satellite of China that flew in the space for 18 days in 2004. These space-exposed seeds were planted alongside the control (WT) Baron seeds in the field. There was no observable difference in the F1 plants. The seed heads of F1plants were collected and seeded in the field. Several dwarf plants were found in the F2 generation. Single seed heads from the F2 plants were collected and seeded separately in the field. The two dwarf selections, A12 and A16, were obtained from the F3 dwarf plants. Compared to the WT Baron, both A12 and A16 exhibited slower rate of growth, darker leaf color and wider leaf blade (Fig 1A). To test whether exogenous GA could rescue the plant height of the dwarf mutants, GA_3_ solution at 150 ppm was foliar-sprayed on the dwarf mutant plants until runoff was detected. Plant heights were measured before and 7 days after GA_3_ spraying.

### Sample preparation and sequencing

Individual grass seed from F2 of each mutant was planted in SC-10 single cell 3.8 cm diameter × 21 cm deep containers. The mature leaves were used for the sequencing studies. The soil mixture was 1:1:1 ratio of sand, soil and perlite. Kentucky bluegrass plants were watered and fertilized (20N-10P-20K soluble) as needed.

Kentucky bluegrass leaf blades were collected from WT, A12 and A16 with three replicates to minimize transcriptional variation introduced by changes in the environment, growth stage or physiological differences of the plants. Leaf tissue was collected for RNA extraction and immediately frozen in liquid nitrogen. Total mRNA was extracted using Trizol kit (Beijing Ding Guo Chang Sheng Biotechnology) according to the manufacturer’s instructions (the electrophoresis gel figure of RNA was shown in the S1 Fig), and the cDNA library construction and normalization were performed as described previously [24]. Total RNA samples were prepared for sequencing in a full run on the Illumina HiSeqTM2000 platform.

### Sequencing, assembly, and library annotation

A strict quality-filtering pipeline was used to select reads for assembly. Since there is no reference Kentucky bluegrass genome, cleaned and qualified reads were assembled de novo in Trinity (v2012-10-5 http://trinityrnaseq.sourceforge.net/), which has been shown to perform the best for restoring full-length transcripts as described previously [25]. The sequences with >95% similarity were grouped into one class, and the longest sequence of each class was treated as a unigene during later processing. The full-length reads were directly mapped to the reference transcriptome libraries using the RSEM (v 0.7) alignment [26]. Mapping parameters allowed for only the uniquely mapped reads with two mismatches.

Taxonomic and functional annotation of the transcripts was conducted using Blast2GO [27] software to run BLASTx and BLASTn algorithms against non-redundant nucleotide/protein (NR) database from the National Center for Biotechnology Information (NCBI, National Institutes of Health). Combined graphs for GO classification associated with statistically transcripts are presented in the results section.

### Transcript expression analysis

Read counts of transcripts with a reciprocal match to the reference transcriptome were counted and extracted for gene expression analysis. Due to the polyploidy of the genomes, and a high number of closely related paralogues within plants, a portion of aligned reads will align to more than one transcript. These ‘multi-mapped’ reads can lead to false read counts for many transcripts. Therefore, the three replicates within A12, A16 and WT were treated for statistical analysis. FPKM (expected number of Fragments Per Kilo base of transcript sequence per Million base pairs sequenced) values were calculated for each transcript by dividing the number of mapped reads by the transcript length and the number of total sequenced reads in this library. The matrix of read counts was input into DESeq R statistical package to identify transcripts with significant expression differences among A12, A16 and WT (FDR <0.05) [28]. Transcripts with at least 2-log fold change in transcript abundance were regarded as up-regulated.

The log-fold FPKM values of selected transcripts were validated by real time reverse transcription polymerase chain reaction (RT-PCR) with ABI PRISM 7700. RNA was extracted from WT, A12 and A16 (3 replicates each) using the Trizol kit and treated with RQ1 DNase (Promega, St. Louis Obispo, CA) according to the manufacturer’s instructions, with RNase Inhibitor (Promega) added to each 100 μL of RNA extract. Complementary DNA was created from 1 μg of total RNA using M-MLV (TOYOBO) reverse transcriptase. PCR was conducted using the SYBR supermix (Genview) per manufacturer’s instructions. The primer pairs were shown in the S1 Table. The cycle conditions were 95°C for 2 minutes followed by 40 cycles of 95°C for 30 s, 55~60°C for 30 s, and 72°C for 30 s. The melting temperature profiles and gel electrophoresis were used to evaluate the specificity of the reactions and the absence of primer dimers. Three technical replicates of each cDNA sample were used in each experiment.

### Identification of SSRs, SNPs and InDels

Assembled transcripts with coverage of at least four reads were screened for simple sequence repeats (SSRs), single nucleotide polymorphisms (SNPs), insertions and deletions (InDels) using Misa and GATK softwares [29]. The minimum repeat-unit size for di-nucleotides was set at six and five for tri- to hexa-nucleotide repeats.

### Determination of gibberellin

Grass leaves were collected, weighted, immediately frozen in liquid nitrogen, and stored at -80°C. To extract phytohormones, plant samples (5 mg) were frozen in liquid nitrogen and finely ground into powder followed by extraction with 500 μL modified Bieleski solvent (methanol/H_2_O, 80/20, v/v) at 4°C for 12 h. The test samples were prepared as previously described and then analyzed on the system of Nano-LC–ESI-Q-TOF-MS (Kyoto, Japan) [30].

## Results

### Sequencing and assembling

**Fig 2:**
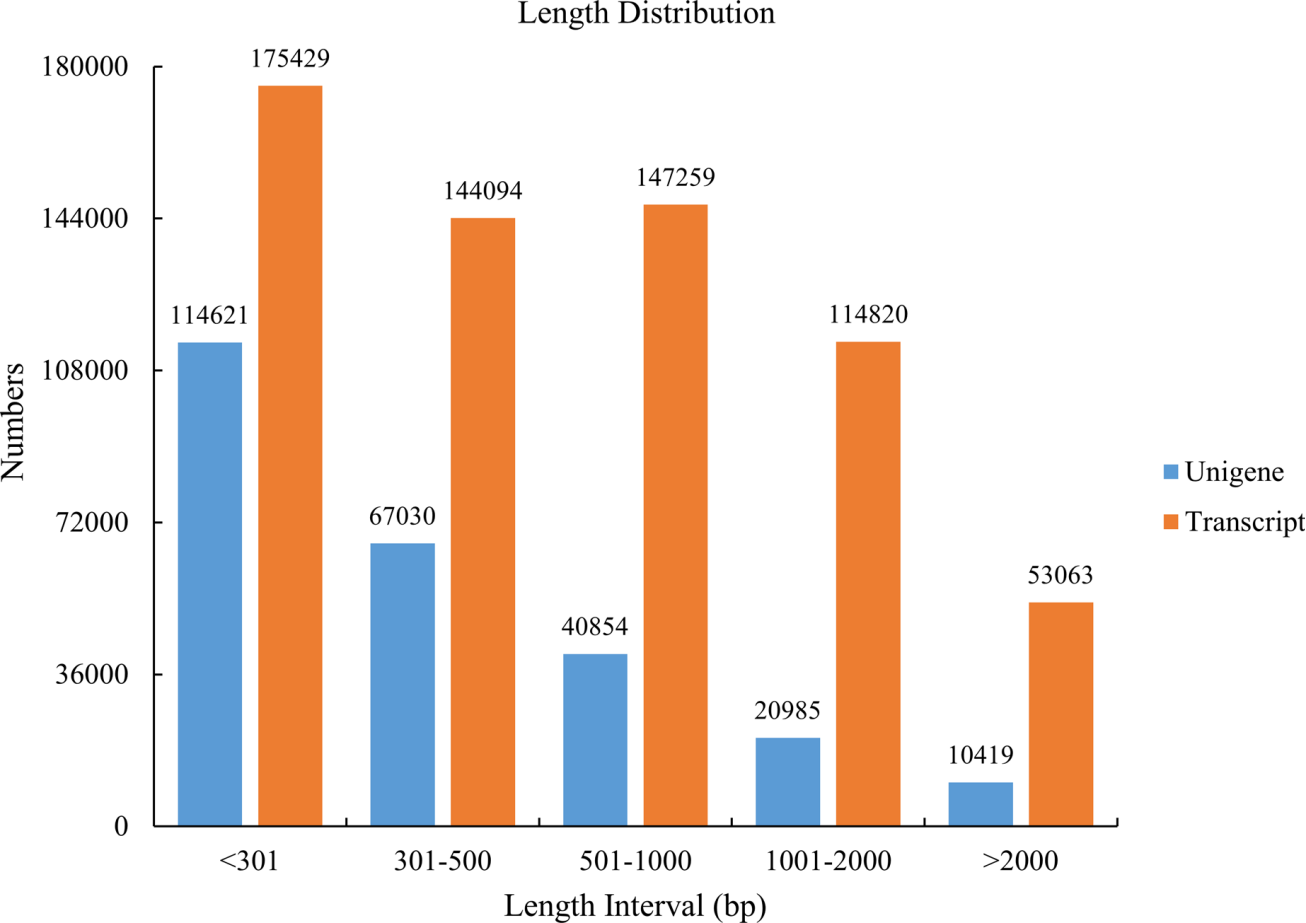
*De novo* assembly length distribution. Histogram of the sequence-length distribution of transcripts and unigenes.

Using Illumina technology, a total of 555,355,250 paired-ends reads were produced for three replicates of WT, A12 and A16, which generated 45 Gb of data. After a trimming process to remove adaptor and primer sequences, poly-A tails as well as short, long and low quality sequences, 531,213,726 cleaned and qualified reads were *de novo* assembled and produced a set of 634,665 transcripts using Trinity (Table 1). More than half of the total assembled transcripts were > 700 bp (N50 = 1305) in length. We selected 253,909 sequences (40.07% of the total transcripts) as unigenes, with a mean length of 560 bp and an N50 of 777 bp. The length distribution of the transcripts and the unigenes are shown in Fig 2.

**Table 1:** Summary on assembled transcripts and unigenes of all Kentucky bluegrass samples.

Total raw reads	555,355,250
Total clean reads	531,213,726
Q20 bases (%)	96.08
GC content (%)	54.34
Total transcripts	634,665
Total length of transcripts (bp)	519,828,684
Transcripts with N50 length (bp)	1,305
Transcript mean length (bp)	819
Total unigenes	253,909
Total length of unigenes (bp)	142190,206
Unigenes with N50 length (bp)	777
Unigenes mean length (bp)	560

### Functional annotation of the transcriptome

**Fig 3:**
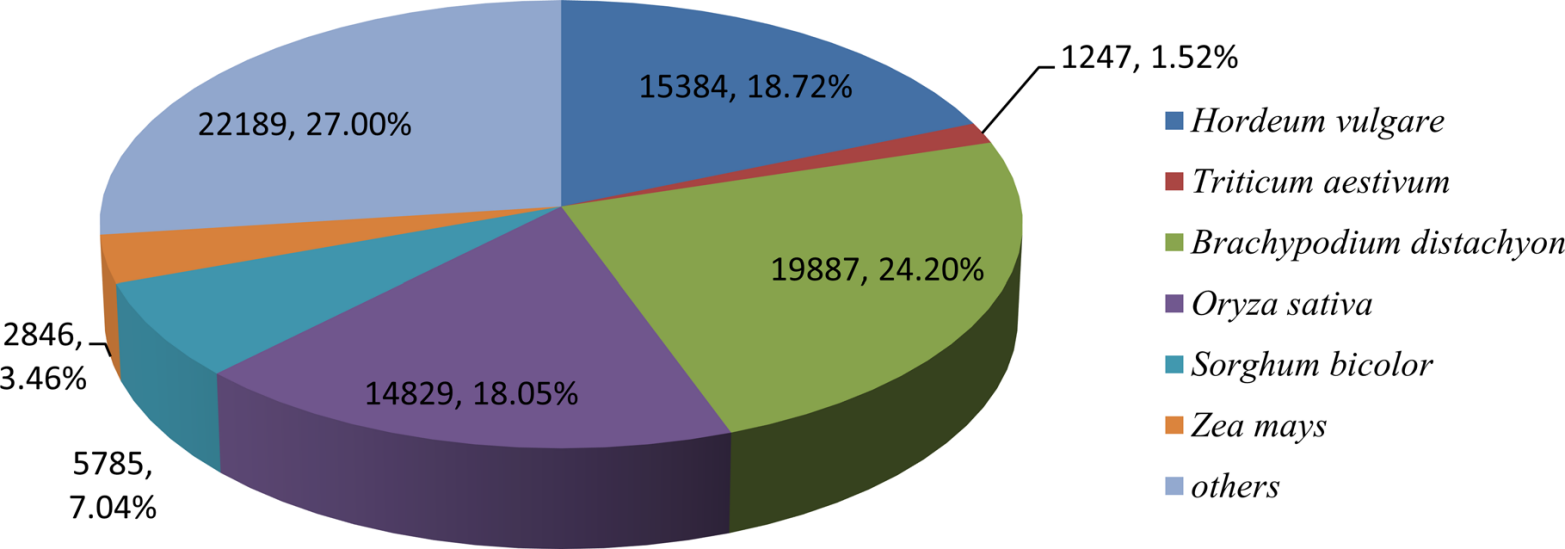
Summary and taxonomic source of BLASTx matches for Kentucky bluegrass unigenes. Percentage of unique best BLASTx matches of unigenes grouped by genus.

In this study, the Kentucky bluegrass 253,909 assembled unigenes were queried against seven protein databases, 82,171 sequences (32.36%) were found to be similar to proteins in the NR database, and 66,707 sequences (26.27%) were similar to those in the Gene Ontology (GO) database (Table 2). Further investigation showed that among the NR BLASTx top hits, Kentucky bluegrass unigenes were significantly similar to *Brachypodium distachyon* proteins (19,887, 24.20%), followed by *Hordeum vulgare* (15,384, 18.72%) and *Oryza sativa* (14,829, 18.05%) (Fig 3). The other unigenes (22,189, 27.00%) were similar to proteins of important turfgrass or forage grasses, including *Lolium perenne*, *Festuca arundinacea*, *Dactylis glomerata* and *Aegilopsovata*.

**Table 2:** Annotation summary of 253,909 unigenes of Kentucky bluegrass.

Database	Hits	Hits percentage (%)
NR	82,174	32.36
NT	13,976	5.5
SwissProt	42,691	16.91
KO	15,573	6.13
GO	66,707	26.27
KOG	10,628	4.18
PFAM	55,136	21.71
All Databases	2,097	0.82

**Fig 4:**
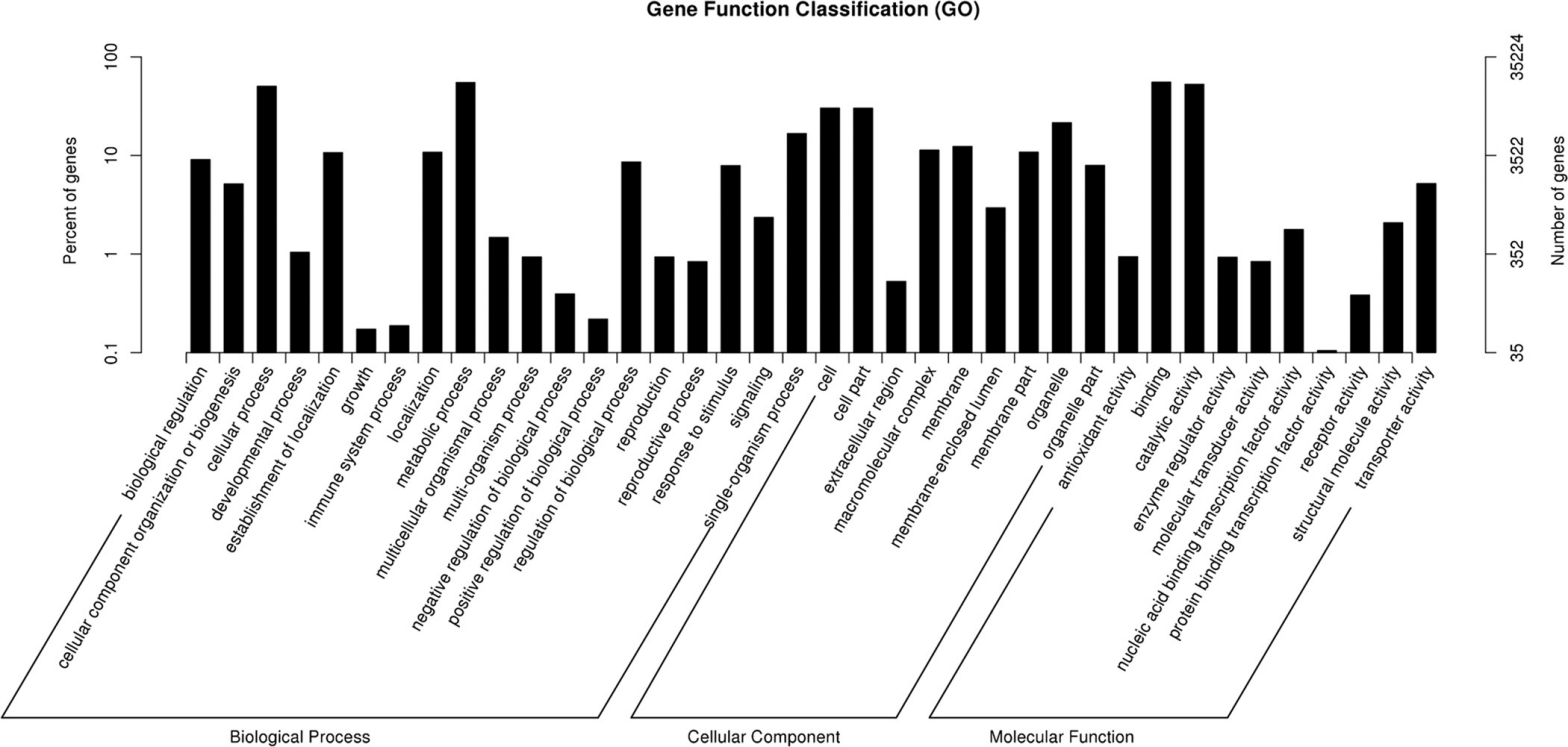
Gene Ontology (GO) distributions for the Kentucky bluegrass transcriptome. The main functional categories in the biological process, cellular component and molecular functions found in the transcriptome are related to plant physiology. The ordinate indicates the number of unigenes. Bars represent the numbers of assignments of Kentucky bluegrass proteins with BLASTx matches to each GO term. One unigene may be matched to multiple GO terms.

A homology search against the UniProtKB/SwissProt (UniProt Knowledgebase) database which contains a small number of proteins produced 42,691 hits (16.91%) (Table 2). The unigenes were annotated with gene names and Gene Ontology (GO) terms based on sequence comparisons between Kentucky bluegrass (*Poa pratensis*) unigenes and the NR database. The distribution of protein assignments to specialized GO terms indicates that the Kentucky bluegrass sequences represent proteins from a diverse range of functional classes (Fig 4).

**Fig 5:**
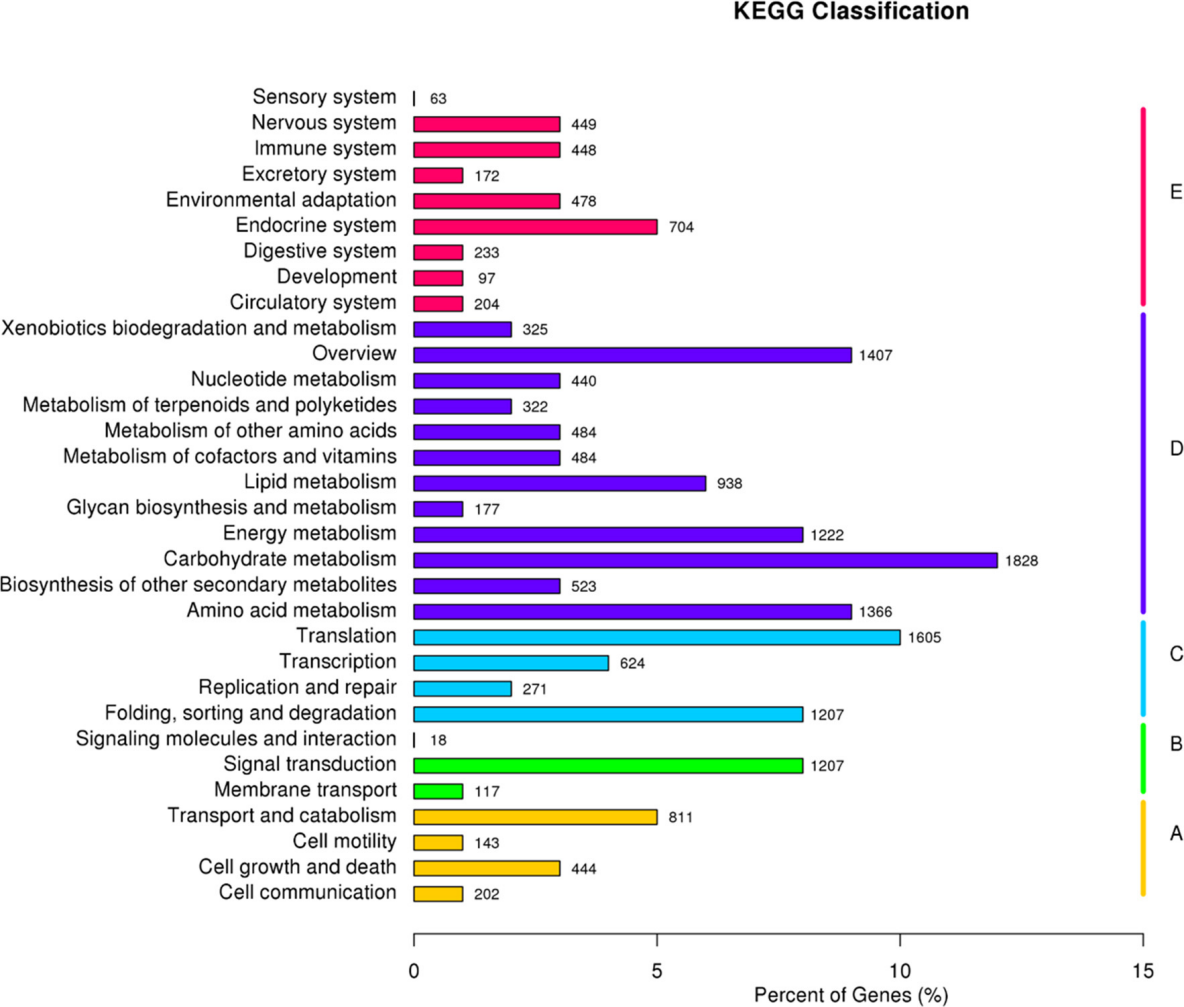
KEGG classification of the Kentucky bluegrass transcriptome. Unigenes involved in the metabolism pathways by KEGG classification are divided into five groups: A. Cellular processes; B. Environmental information processing; C. Genetic information processing; D. Metabolism; and E. Organismal systems.

To correlate Kentucky bluegrass unigenes with known metabolic pathways, the KAAS server was used to assign sequences with KEGG orthology (KO) terms and their respective KEGG maps [31] [32]. A total of 15,573 (6.13%) assembled unigenes were associated with 32 pathways (Fig 5).

### Putative marker discovery

**Fig 6:**
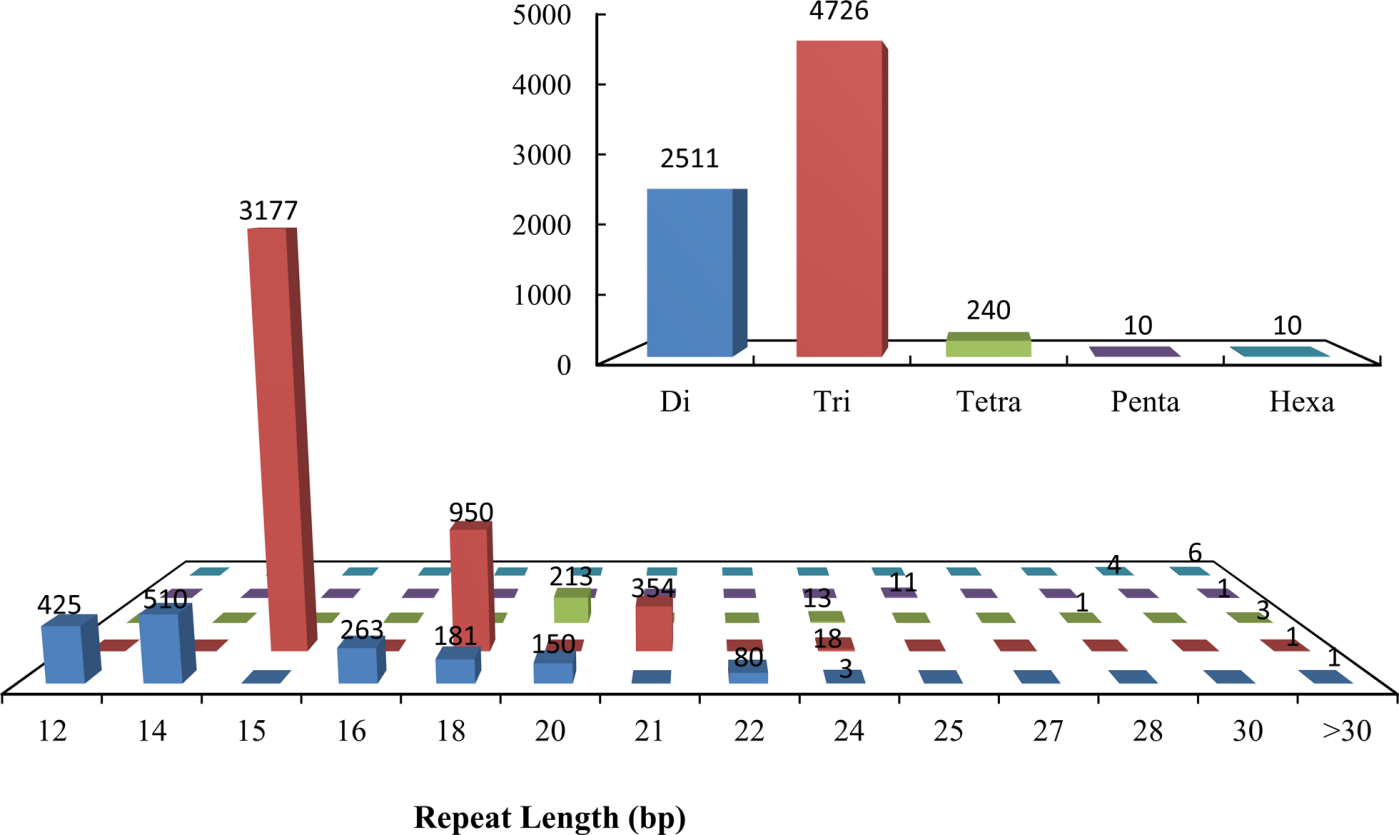
Distribution of simple sequence repeats (SSRs) in Kentucky bluegrass expressed sequence tags (ESTs). Di-, tri-, tetra-, penta- and hexa-nucleotide repeats were analyzed and their frequencies were plotted as a function of the repeat number. The upper right histogram shows the distribution of the total number of SSRs in different classes.

Transcriptome sequencing provides valuable information for development of molecular markers [32]. The large data set generated by transcriptome sequencing makes it possible to identify different types of polymorphisms and putative markers, such as SNPs, SSRs and microsatellites [33]. Based on the analysis of all assembled unigenes, Di- to hexa-nucleotide SSRs with a minimum repeat unit size of five (for tri- to hexa-nucleotide) or six (for di-nucleotide) were detected in all transcripts. A total of 14,174 SSRs were identified, and the different repeat types were determined. Among the various classes of SSRs, the most abundant repeats were the tri-nucleotides, with 63.0%, followed by di-nucleotides with 33.50% (Fig 6). There are 834,342 SNPs and 105,497 InDels identified from the sequenced unigenes. If validated, these markers will facilitate studies on population genetics, linkage mapping and comparative genomics of Kentucky bluegrass.

### Comparison of FPKM ratios to relative transcription ratios

**Fig 7:**
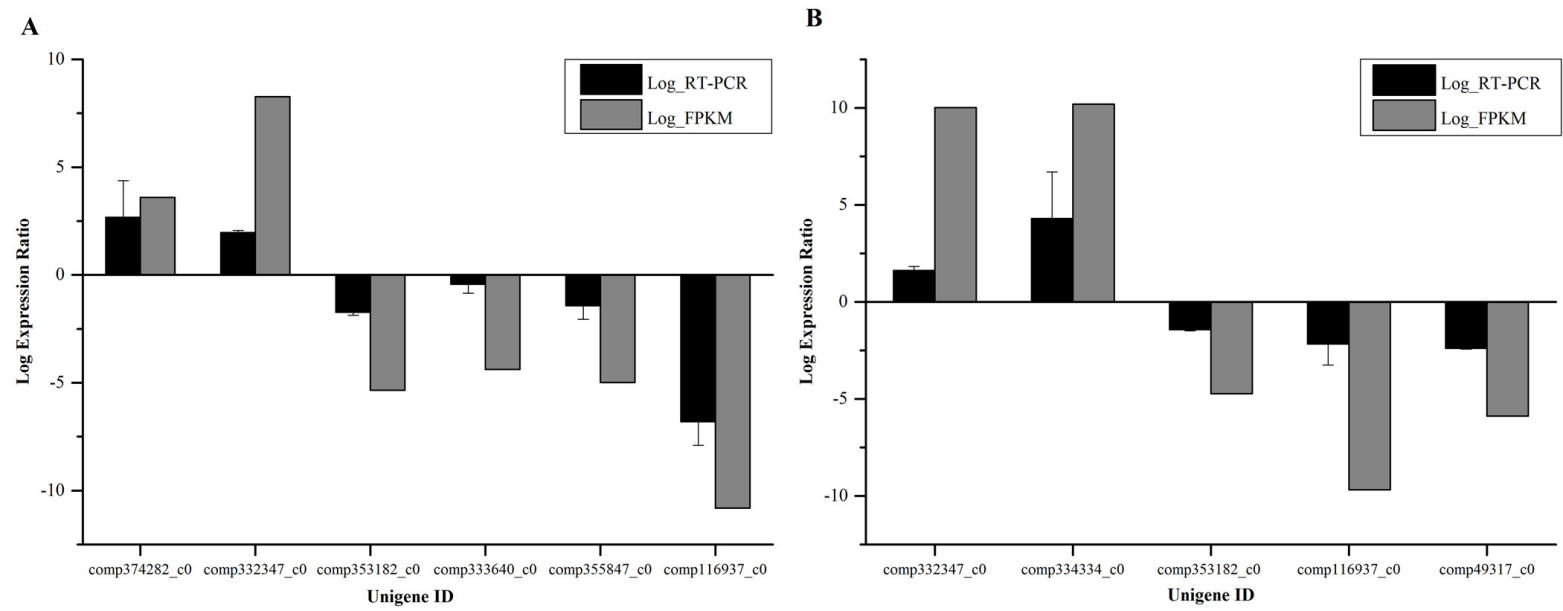
**A, Validation of FPKM data for the selected A16 and WT transcripts using real time RT-PCR for relative gene expression. B, Validation of FPKM data for the selected A12 and WT transcripts using real-time RT-PCR for relative gene expression.** Bars show means and standard errors of three biological replicates.

Clean reads were mapped to the assembly transcriptome using Bowtie [34], and their respective read abundances were estimated by RSEM (RNA-Seq by Expectation-Maximization) [26]. FPKM (expected number of Fragments Per Kilo base of transcript [35] sequence per Million base pairs sequenced) value was calculated for all samples and the Log_2_ ratio was calculated to reflect the fold changes compared with WT. Relative transcription data from real-time RT-PCR assays were obtained for selected unigenes with significantly different transcription levels among the different samples sequenced. The relative transcriptional data confirmed the accuracy of the FPKM calculation and statistical analysis (Fig 7).

### Differentially expressed genes analysis

**Fig 8:**
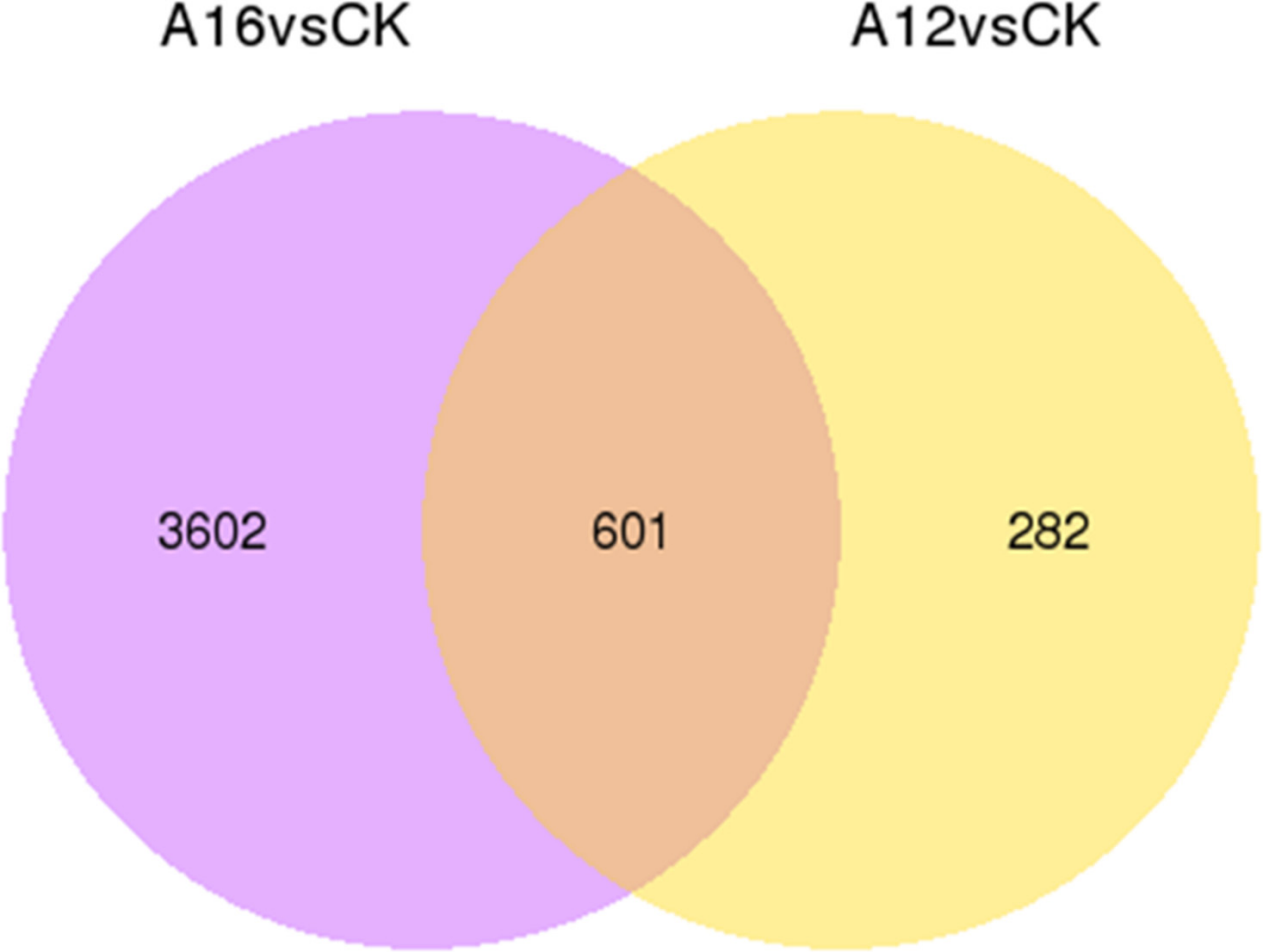
Venn diagram of differentially expressed genes. The numbers in each circle represent the total number of different genes in the compared combination, and the overlapping part is the shared different genes between two combinations.

**Fig 9:**
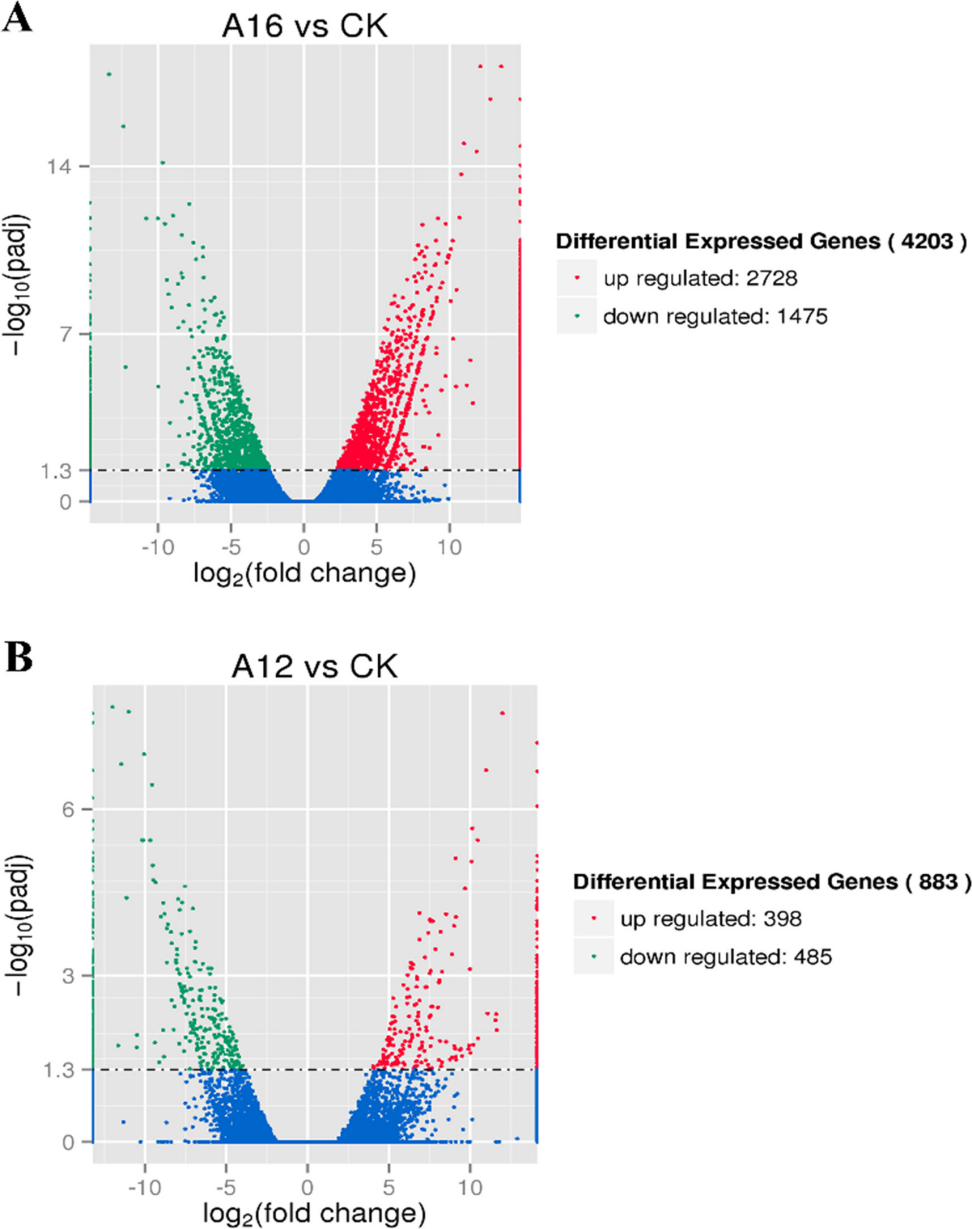
Volcano plots of differentially expressed sequences. The abscissa represents the expressed fold change of genes in different samples. The ordinate represents the statistically significant difference degree. The lower *p*-adj and higher -log_10_ (*p*-adj) values represent greater differences. The scatters in diagram stand for each gene, the blue dot indicates there was no significant difference of genes. The up-regulated genes were represented by a red dot, down-regulated genes by a green dot.

Using the DESeq Bioconductor package [35], a FPKM cut-off value of 0.5 was employed to define the unigenes expressed in sequenced samples [36], and the expression levels of the selected reciprocal hits were analyzed for statistical significance. P-value < 0.05 was adjusted for multiple testing in this study. Based on these values, the unigenes were determined to be unique or shared with the compared combinations (A16 vs WT, A12 vs WT), as shown in Fig 8. In brief, there were 883 differentially expressed genes (DEGs) between WT and A12 libraries, and 4,203 DEGs between WT and A16 libraries, and the expression levels of 601 DEGs in all three libraries. Furthermore, the up-regulated or down-regulated genes were filtered by the log_2_ fold change of ⩾1 and the *p*-value adjusted (*p*-adj)<0.005to avoid false positives, as shown by the volcano plots in Fig 9.

KO and GO terms were assigned to all differentially expressed genes between A16 vs WT and A12 vs WT libraries, including those incomplete alignments to a reciprocal sequence. Transcripts with statistically significant changes of FPKM values were determined if the selected DEGs were patterns of transcriptional pathway with increase or decrease in gene expression [37]. DEGs between A12 and WT as well as A16 and WT were summarized in Tables 3 and 4, respectively, which showed clearly that the DEGs are mainly in the pathways of biosynthesis of terpenoids, plant hormones and signal transduction, chlorophylls metabolism and flavonoid biosynthesis. The differential pathways between the two compared combinations (A12 vs WT, A16 vs WT) are almost overlapping (Tables 3 and 4). However, the different fold change values indicate that there is diversity between the dwarf mutants A12 and A16. The data demonstrate that the differences of these pathways might cause the different physiological and morphological performances between dwarf mutants and WT plants, which deserve further investigation.

**Table 3:** The list of significantly expressed genes between A12 and WT plants.

Transcript types	Unigene ID	Unigenes length(bp)	Log2 fold change	P adjusted value	Description
Terpenoid biosynthesis	comp353182_c0	1136	-4.7232	0.014512	1-deoxy-D-xylulose-5-phosphate synthase 2
	comp330141_c0	603	-4.8627	0.017326	1-deoxy-D-xylulose-5-phosphate synthase
	comp351187_c0	1318	-4.303	0.036121	1-deoxy-D-xylulose-5-phosphate synthase
	comp310821_c0	878	Inf	0.0018751	IPP transferase
Plant hormone synthesis and signal transduction	comp62710_c0	436	-7.742	0.0023938	DELLA protein SLN1
	comp116937_c0	961	-9.68	3.64E-06	auxin binding protein
Chlorophyll metabolism	comp332347_c0	1177	10.015	0.024825	chlorophyllase-1-like
Flavonoid biosynthesis	comp321596_c0	1794	10.976	1.98E-07	flavanone 3-hydroxylase

**Table 4:** The list of significantly expressed genes between A16 and WT plants.

Transcript types	Unigene ID	Unigenes length(bp)	Log2 fold change	P adjusted value	Description
Terpenoid biosynthesis	comp353182_c0	1136	-5.3513	2.8164E-06	deoxyoxylulose-5-phosphate synthase
	comp265264_c0	1069	Inf	0.025575	isopentenyl transferase
	comp349265_c0	1358	2.8875	0.040376	squalene epoxidase
chlorophyll metabolism	comp332347_c0	1177	8.2673	0.0051754	chlorophyllase-1-like
Plant hormone synthesis and signal transduction	comp355847_c0	2846	-4.9866	0.042206	gibberellin 3-beta-dioxygenase 4-like
	comp62710_c0	436	-4.4192	0.00038555	DELLA protein SLN1
	comp116937_c0	961	-10.806	1.5024E-12	auxin binding protein
	comp333640_c0	1290	-4.3805	0.013885	probable auxin efflux carrier component 5-like
Carotenoid biosynthesis	comp234423_c0	867	Inf	0.031879	9-cis-epoxycarotenoid dioxygenase 1
Flavonoid biosynthesis	comp365174_c0	2648	-4.3937	0.043267	flavonoid 3'-monooxygenase
	comp321596_c0	1794	9.0426	1.2225E-10	flavanone 3-hydroxylase

## Discussion

The available genetic resources are limited for Kentucky bluegrass. The aim of this research was to expand Kentucky bluegrass genetic resource and characterize the differences in unigenes between WT and the dwarf mutants induced by space mutation. In the present study, employing the Illumina sequencing platform, 253,909 unigenes were reconstituted for the WT and two dwarf mutant selections of Kentucky bluegrass. Differentially expressed unigenes were found between WT and dwarf mutantsA12 and A16.

Comparing to the NR database, 24.2% of the transcripts had BLASTx hits to Brachypodium (*Brachypodium distachyon*) proteins. This indicates that Kentucky bluegrass is mostly related to Brachypodium, a cool season, C_3_ grass that is often regarded as a model plant for grass studies [38]. Compared to barley (*Hordeum vulgare*), Kentucky bluegrass had 18.72% BLASTx hits (Fig 2). Therefore, we speculate that the closely related species to Kentucky bluegrass are Brachypodium and barley.

Additionally we examined the morphological differences among WT, A12 and A16. The main differences in morphology among them are in plant height (WT>A12>A16), leaf blade color and width (A16>A12>WT). Previous researches suggested that plant hormones play an important role in plant growth and development, especially in dwarfing phenotype [39, 40]. For instance, GA-sensitive dwarf mutants were found in many plants, such as Arabidopsis [41], wheat [42], barley [5], rice [43] and others [44–47]. Some studies showed brassinolide and auxin might also interfere with plant growth and cause dwarf phenotype [46, 47]. The chlorophyll and carotenoids synthesis pathway might be critical in leaf color [48]. The differences in gene expression levels between WT and A12, as well as WT and A16 may account for their phenotypic differences. Therefore, it is critical to select differentially expressed genes based on the GO and enriched KEGG terms for hormone and chlorophyll synthesis to further our understanding of plant dwarfing and leaf blade color (Tables 3 and 4).

### The differentially expressed genes in MEP pathway

Terpenoid metabolism is one of the important pathways for secondary metabolites in plants [49]. The metabolic route and its end products will determine plant growth, photosynthesis, respiration and resistance to stress etc. [50]. The 2-C-methylerythritol 4-phosphate (MEP) pathway supplies precursors for plastidial isoprenoid biosynthesis including carotenoids, the side chain of chlorophylls and plant hormones, which are derived from the common precursor isopentenyl diphosphate (IPP). The first enzyme in the MEP pathway, 1-deoxy-D-xylulose 5-phosphate (DXP) synthase (DXS), is considered to be essential in the control of plastidial isoprenoid production [51]. Our results demonstrated that the *dxs* gene shows genetic difference between A12 and WT as well as A16 and WT (Tables 3 and 4). Furthermore, the three differential *dxs* unigenes (comp353182_c0, comp330141_c0 and comp351187_c0) are homologous to *Brachypodium distachyon* genes. Taking comp353182_c0 as an example, the putative 1-deoxy-D-xylulose-5-phosphate synthase 2 (*dxs*2) gene was down-regulated in both A12 and A16, but the fold changes of down-regulation were 4.7232 and 5.3513, respectively. This indicates that the two dwarf mutants were not only obviously different from the WT, but also different from each other.

In addition, some differentially expressed genes encode enzymes in the branch of MEP pathway, such as isopentenyl transferase (comp265264_c0) and IPP transferase (comp310821_c0). The squalene epoxidase (SE) (comp349265_c0) was up-regulated in the A16 dwarf mutant. SE, a monooxygenase [52], is one of the important enzymes in the triterpenoid biosynthetic pathway. It catalyzes the formation of 2, 3-oxidosqualene, the precursor of sterol and sesquiterpenoids which are critical in plant growth and development and in disease resistance [53].

### DEGs in plant hormone synthesis

One of the plant dwarfing mechanisms involves the blocked metabolism and signal transduction pathways of plant hormones, such as gibberellins (GA) or auxin (IAA). The total endogenous GA content of WT, A12 and A16 was measured by Nano-LC-ESI-Q-TOF-MS analysis [30]. Our results showed that the GA concentration in WT was 9.20 ng.g^-1^F.W., which was higher than that in A12 (8.09ng.g^-1^F.W.) and A16 (5.43ng.g^-1^F.W.), indicating that the GA synthesis might be blocked in the dwarf mutants (Table 5).

**Table 5:** The level of GAs among three samples.

Analyte	GA_3_	GA_5_	GA_12_	GA_19_	GA_53_	Total
WT	0.14±0.02	0.91±0.06	2.69±0.27	4.86±0.38	1.00±0.06	9.6
A12	0.30±0.04	1.01±0.06	1.01±0.12	5.21±0.94	0.56±0.02	8.09
A16	0.27±0.04	0.73±0.14	1.00±0.07	2.83±0.20	0.60±0.05	5.43

The values in the table represent means±SD and the unit is ng.g-1F.W. Additionally, GA_1_, GA_4_, GA_7_, GA_15_, and GA_20_ were not detected.

As mentioned above, the end products of the MEP pathway provide the precursor for plant hormones (GA, BR, cytokinins and abscisic acid) [54, 55]. Therefore, the down-regulation of the upstream *dxs* unigene might lead to the down-regulation of the downstream genes and enzymes, especially the metabolic pathway of GA, thus affecting the plants’ physiological and morphological performances. Additionally, there might be other mutations in key genes in these pathways at the final synthesis of terpenoids, which could result in the variation of hormone content and plant physiology. For example, our data showed that the gene encoding gibberellin 3-beta-dioxygenase 4-like (comp355847_c0) in the branch of the GA synthesis was differentially expressed. The function of this gene needs to be further studied.

Our data showed that some DEGs related to the synthesis of auxin, including auxin binding protein (comp116937_c0) and auxin efflux carrier component 5-like (comp333640_c0), were down-regulated in the dwarf mutants. It is known that there is synergy or antagonism among hormones in plants. For instance, auxin is known to up-regulate the transcription of GA3ox1 and down-regulate that of GA2ox in pea [56]. Miyawaki *et al*. (2004) showed that some of the phosphate-isopentenyltransferase genes, which are vital in cytokinin biosynthesis, are up-regulated by auxin in Arabidopsis [57]. Hence, besides the mutation of related genes, the interaction of various hormones can lead to the variation of hormone content and the physiological differences.

Moreover, after exogenous GA_3_ application, the dwarf plants (A12 and A16) were recovered to the wild type in height as shown in Fig 1B and 1C. Based on the transcriptome data and the physiological performance, we speculate that the dwarfing of our Kentucky bluegrass may be related to differentially expressed *dxs* genes and DEGs in hormone synthesis, especially in the GA and IAA biosynthetic pathways. However, further exploration is necessary to verify this speculation.

## Conclusions

This study presents the first comprehensive transcriptome data and analysis of gene function of Kentucky bluegrass (*Poa pratensis* L.), which is the most extensively expressed sequence resource available for Kentucky bluegrass up to date. A total of 253,909 unigenes were identified and 14,174 SSR and 939,839 SNP/Indel loci were detected. The GO and KEGG analysis were carried out, and all unigenes were classified into functional categories with the aim of understanding their functions and metabolic pathways. These data can be used to develop oligo-nucleotide microarrays or serve as a reference transcriptome for future studies in large-scale gene expression assays. Meanwhile, the differentially expressed genes between dwarf mutants and WT provide abundant information to investigate the possible mechanisms of plant dwarfing, which will facilitate our understanding of genetic variation in populations and the genetic control of crucial traits in Kentucky bluegrass. Furthermore, such large-scale sequence data is a potentially valuable scientific resource for genetic mapping, marker-assisted breeding in Kentucky bluegrass and comparative genome analysis for Pooideae plants.

## Supporting Information

S1 Fig The electrophoresis gel figure of RNA for 9 samples. (JPG)

S1 Table The primer pairs for real time reverse transcription polymerase chain reaction. (XLSX)
