# Correction: *De Novo* Transcriptome Analysis for Kentucky Bluegrass Dwarf Mutants Induced by Space Mutation

**DOI:** 10.1371/journal.pone.0155452

**Published:** 2016-05-06

**Authors:** Lu Gan, Rong Di, Yuehui Chao, Liebao Han, Xingwu Chen, Chao Wu, Shuxia Yin

In Fig 1, Fig 1B was incorrectly duplicated as Fig 1A. Please view the correct Fig 1 here.

**Fig 1:**
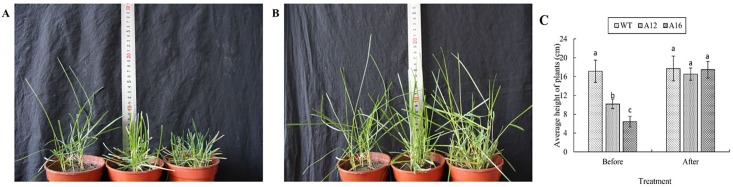
Plant height comparison before and after GA_3_ spraying. A. WT, A12 and A16 (from left to right) plants without GA_3_ spraying. B. WT, A12 and A16 (from left to right) plants 7 days after foliar-spraying with GA_3._ C. Plant height comparison between WT, A12 and A16 before and after GA_3_ spraying. Vertical bars on the top indicate standard deviation, and bars with different letters indicate significant difference (*p* < 0.05).
