# Supplementary figures and images for: De Novo Transcriptome Analysis for Kentucky Bluegrass Dwarf Mutants Induced by Space Mutation
Source: PLoS One. 2016 Mar 24;11(3):e0151768. doi: 10.1371/journal.pone.0151768 (PMC4807101; doi:10.1371/journal.pone.0151768)

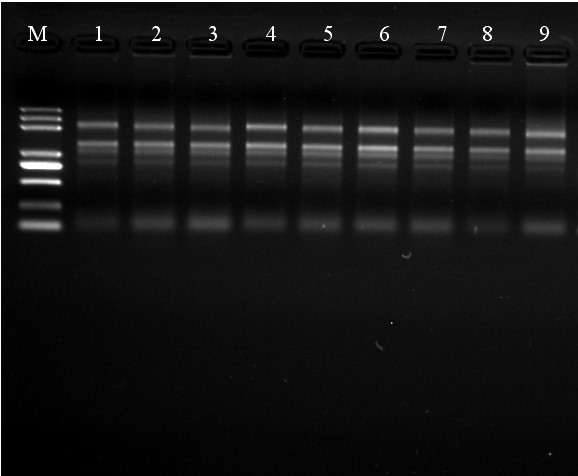

Supplement: S1 Fig — (JPG) [file pone.0151768.s001.jpg]
